# Toward Capturing Momentary Changes of Heart Rate Variability by a Dynamic Analysis Method

**DOI:** 10.1371/journal.pone.0133148

**Published:** 2015-07-14

**Authors:** Haoshi Zhang, Mingxing Zhu, Yue Zheng, Guanglin Li

**Affiliations:** The Key Laboratory of Human-Machine Intelligence-Synergy Systems, Shenzhen Institutes of Advanced Technology (SIAT), Chinese Academy of Sciences, Shenzhen, Guangdong, PR China; Temple University, UNITED STATES

## Abstract

The analysis of heart rate variability (HRV) has been performed on long-term electrocardiography (ECG) recordings (12~24 hours) and short-term recordings (2~5 minutes), which may not capture momentary change of HRV. In this study, we present a new method to analyze the momentary HRV (mHRV). The ECG recordings were segmented into a series of overlapped HRV analysis windows with a window length of 5 minutes and different time increments. The performance of the proposed method in delineating the dynamics of momentary HRV measurement was evaluated with four commonly used time courses of HRV measures on both synthetic time series and real ECG recordings from human subjects and dogs. Our results showed that a smaller time increment could capture more dynamical information on transient changes. Considering a too short increment such as 10 s would cause the indented time courses of the four measures, a 1-min time increment (4-min overlapping) was suggested in the analysis of mHRV in the study. ECG recordings from human subjects and dogs were used to further assess the effectiveness of the proposed method. The pilot study demonstrated that the proposed analysis of mHRV could provide more accurate assessment of the dynamical changes in cardiac activity than the conventional measures of HRV (without time overlapping). The proposed method may provide an efficient means in delineating the dynamics of momentary HRV and it would be worthy performing more investigations.

## Introduction

Heart rate variability (HRV), the variation of the period between consecutive heart beats over time is the result of complex regulation mechanism through which the autonomic nervous system (ANS) rules heart rate and keeps cardiovascular parameters within physiological ranges [1]. HRV analysis provides significant non-invasive information on ANS activity [2,3]. Many studies have demonstrated the significant relationships between the HRV and physiological and psychological activity such as myocardial infarction, mental stress and anxiety disorders [3–5]. A number of methods have been used to evaluate the variation of instantaneous heart rate, most of which are derived from time-domain analysis and frequency-domain analysis of RR intervals in electrocardiograph (ECG) [1–3, 6–12]. For the time-domain analysis of HRV, statistical and/or geomethrical methods were often used to extract the indice of HRV measures [3]. Various spectral methods such as Hilbert transform [6], wavelet analysis [9–10,12] and time-frequency analysis [7–8, 11] were applied in frequency-domain analysis of HRV. In addition, some non-linear parameters such as approximate entropy were certainly involved in the analysis of HRV [13–14].

Traditionally, almost all analysis methods of HRV carry out over long-term ECG recording (12~24 hours) and short-term ECG recording (2~5 minutes) [3]. Due to the low stability of heart rate modulations during long-term recording, time-domain methods are better than frequency-domain methods for the HRV analysis of long-term recording. For short-term recording, the frequency-domain methods are usually able to provide results that are more easily interpretable in terms of physiological regulations. While the traditional HRV analysis of long-term and short-term recordings provide valuable physiological and pathological information for some cardiological and non-cardiological diseases, for example, myocardial infarction, diabetic autonomic neuropathy, and tetraplegia, and for modification of HRV by some specific intervention, for example, drugs and exercise, it should be note that the averaged measures of temporal and spectral components derived from long-term recordings obscures detailed information about autonomic modulation of RR intervals, and proper assessment of HRV dynamics may lead to substantial improvements in our understanding of both the modulations of heart period and their physiological and pathophsiological corrrelates [3]. On the other hand, in psychological and other related fields, rapid impact on HRV often occur such as the acute increase in heart rate in response to mental stress [5]. When the nature of physiological modulation in heart rate changes from one short-term recording to another or heart rate has a very short time fluctuation caused by pathological or psychological intervention, it is important to catch the changing states of the cardiovascular system as soon as possible. The traditional methods are limited by their inability to adequately assess transient changes in heart rate which are associated with rapid changes in physiological status. A method for measuring momentary HRV (mHRV) that follows changes in cardiac activity is required, so that transient effects on the HRV would be not lost.

The present study aims at proposing a new method to explore temporal changes in heart rate by performing the HRV analysis in a series of consecutive overlapped windowed short segment of ECG recordings. Different from the traditional method that perform computation on discrete segmented recording along with much detail information lost, the overlapped analysis window would introduce much little new data each time with a short period of time increment, which means the time interval between two adjacent measurements is very short, and then the dynamical information would be reflected by the analysis in detail. In addition, the time courses of the measures computed from each analysis window are graphically plotted for continuously monitoring of autonomic nervous function, so that the dynamical interactive cardiac autonomic regulation now can be investigated in real time.

## Materials and Methods

### Data collection

To evaluate the performance of the newly proposed method in delineating the dynamics of mHRV measurement, synthetic time signals and real RR interval series of both human and dog ECG in different physiological status of ANS were applied in the present study.

Several synthetic time signals have been primarily used to assess the performance of the proposed method in evaluating transient changes in heart rate. The most representative one was produced based on the following formula to approximately simulate a significant drift in the frequency location of the respiratory sinus arrhythmia of a RR series during a modulated breathing [8]. The length of the synthetic time series is 20 minutes and its identical time interval is about 1.5 s. The frequency of the signal is changing from high to low, and then to high in a cycle length of 5 minutes.

$$\begin{array}{l} S(n)=\text{cos}(2\pi *n/4-10*pi*\text{cos}(2*pi*n/256)) \end{array}$$ (1)

A group of human RR interval data archived in the MIT-BIH database [15] was used in the study to evaluate the performance of the proposed method in capturing the dynamical interactive cardiac autonomic regulation with HRV analysis. Three cases of the representative RR-interval data series were adopted for the mHRV analysis, which were acquired from healthy young adults during meditation state associated with slow breathing and non-meditation state [16]. The first case was composed of 20-min RR-interval data series, in which the first 10-min data was recorded in a meditation state and the following 10-min data was acquired in a non-meditation state. For the second case, 4-min RR-interval data in non-meditation state was sandwiched between two 13-min meditation data, totally resulting in 30-min series. The third case was composed of the five cascaded segments of RR-interval data series that were 4-min data in meditation state, 2-min data in non-meditation state, 8-min data in meditation state, 2-min data in non-meditation state, and then 4-min data in meditation state, sequentially.

Dog ECG data (S1 Data) was also used to further assess the performance of the proposed method for dynamical analysis of mHRV. The ECG data was collected from a healthy adult beagle dog under an experimental protocol with two successive phases: 1) 30-minute normal sinus ECG recording; and 2) 60-minute autonomic blocking ECG recording with atropine administration (400 mg). Three-lead ECG signals were simultaneously recorded with 4-limb needle electrodes by a commercial multi-channel virtual chart recorder with 12-bit resolution. The sampling frequency was set at 250 Hz to allow the accurate detection and identification of peaks of R-waves. In the present study, only lead-II ECG recordings were used for the HRV analysis.

### Ethics Statement

The dog experiments were approved by the Animal Care Committee (ACC) at Shenzhen Institutes of Advanced Technology, Chinese Academy of Sciences. The ECG data of human were adopted from the MIT-BIH Database which is opened and free-used. The Database was cited according to the requirements of the MIT-BIH Database, so it is unnecessary for us to get an ethic approval for using the ECG data.

### RR Interval Determination and HRV Measures

The QRS complexes in each ECG recording window were determined with a QRS detection algorithm that was adapted from one presented in [17]. In order to convert an irregularly sampled RR interval time series to an evenly sampled signal for frequency-domain analysis, cubic spline interpolation was used to resample the RR interval sequence with a sampling frequency of 4 Hz. Then the resampled RR interval data was de-trended to remove the influence of non-stationarity on the data.

Two commonly used statistical time-domain measures of HRV, standard deviation of the normal-to-normal intervals (SDNN) and square root of the mean squared differences of successive intervals (RMSSD) were adopted in the present study. Intuitively, the SDNN, as the square root of the variance of heart rate time series, reflects the cyclic components responsible for the variability in the RR interval sequence because the variance is equivalent to the total power of the spectrum, and the RMSSD reflects the high-frequency variation components in heart rate [3].

For frequency-domain HRV, three main spectral components are distinguished in a spectrum calculated from short term recordings: very low frequency (VLF: < = 0.04Hz), low frequency (LF: 0.04–0.15Hz), and high frequency (HF: 0.15–0.4Hz) components. The frequency bands of LF and HF are commonly used in HRV analysis since the physiological explanation of the VLF component is much less defined [3, 6]. The Choi and Williams distribution [18], also called exponential distribution (ED) since its exponent kernel, was chosen to implement the joint time-frequency (t-f) transform and power spectral analysis of the RR interval sequence. The exponent kernel minimizes the effect of the cross components without violating the properties of mathematical constraints of the joint t-f distribution. An investigation has shown that the ED distribution is an adequate technique for analysis of heart rate time series with transient spectral evolution as compared with other joint t-f methods [11]. The LF and HF power components of HRV spectrum were derived from the t-f ED of each RR interval sequence. The LF power spectrum, designated as SPLF was calculated at a frequency band of (*LF*_min_, *LF*_max_) = (0.04*Hz*,0.15*Hz*), and the HF power spectrum, designated as SPHF, was calculated at a frequency band of (*HF*_min_, *HF*_max_) = (0.15*Hz*,0.4*Hz*).

For a long-time ECG recording, all the measures were calculated from each RR interval sequence, respectively, resulting in four measure sequences of HRV: [SDNN(t), RMSSD(t), SPLF(t), SPHF(t), t = 1,2,…,T], where SDNN(t), RMSSD(t), SPLF(t) and SPHF(t) are the four measures computed from each segment of a RR interval sequence that derived from the long-time ECG recording by a sliding window, and *T* is the number of ECG segments.

### Overlapped window for HRV analysis

**Fig 1:**
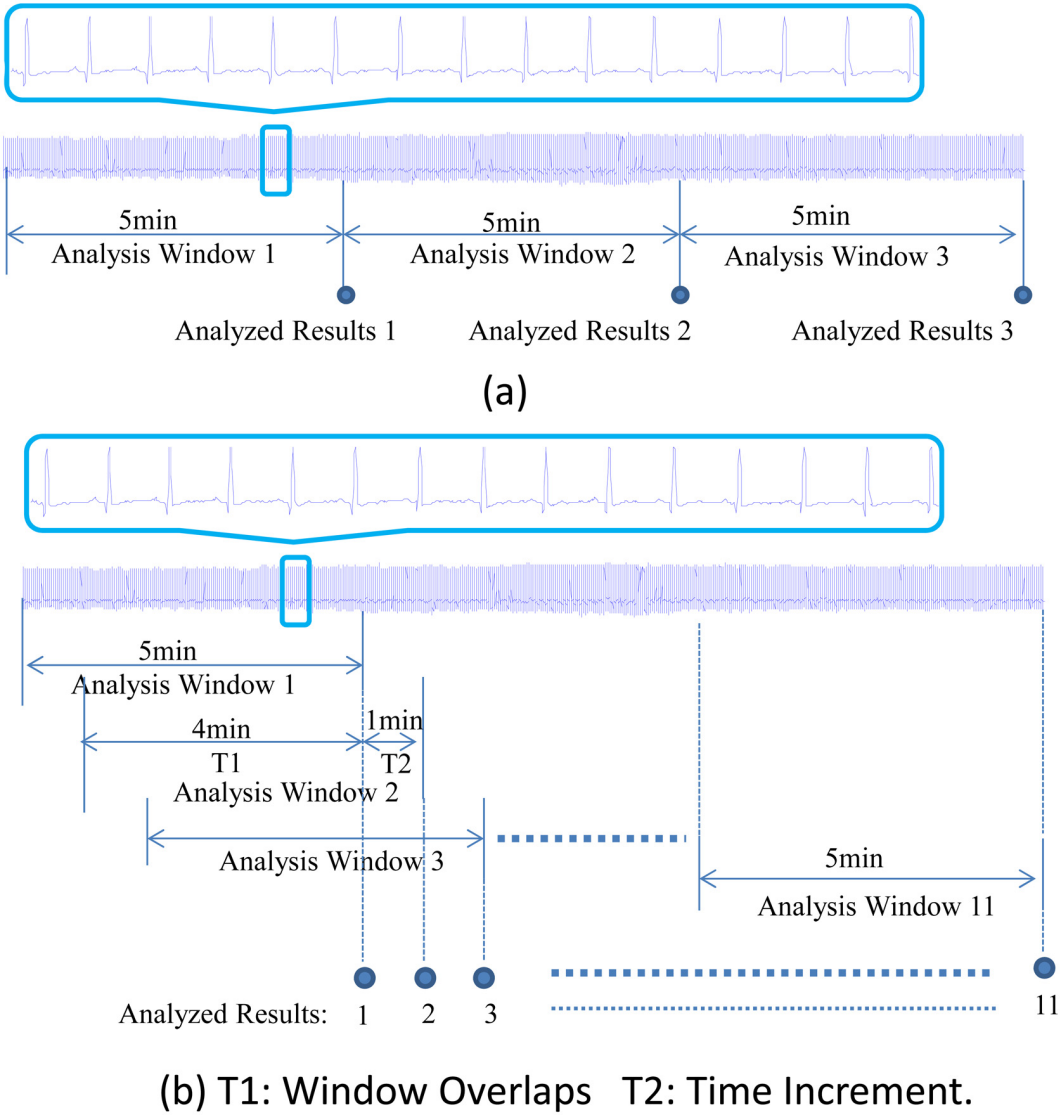
Schematic diagram of the difference between the traditional method and the proposed method with a 1 min time increment. (a) Traditional method of monitoring temporal dynamics of HRV: HRV analysis was performed in each neighbor analysis window, which means the time interval between two analyzed results is 5min, i.e. we can only observe the status changes taking place in the second window at the 10min time point. (b) The proposed overlapping window method of monitoring temporal dynamics of HRV, HRV analysis is performed in each overlapped analysis window.

The HRV analysis was performed on RR intervals extracted from a series of windowed ECG recordings. As 5-min recordings are the standard for short-term analysis of HRV [3], the length of 5-min analysis window was adopted in the study. In the proposed method, the ECG recordings were segmented into a series of 5-min windows with a time increment such as 1 minute (i.e. a 4-min overlapping). The previously mentioned two time-domain parameters and two frequency-domain parameters were obtained from the RR intervals of each 5-min overlapping ECG recordings, resulting in four measure sequences of HRV. In order to investigate the effects of different time increment on capturing the changes of mHRV, six different time increments, 10 s, 30 s, 1 min, 2 min, 4 min, and 5 min were inspected, respectively. Note that for a 5-min window length, a time increment of 5 min means that the neighboring analysis windows have no time overlapping, which would be the case of the conventional HRV analysis and the results of this case could be used as reference for comparison. With a short time increment such as 1 min, the analysis window would introduce 1-min new data each time, which means the time interval between two adjacent measurements is 1 min. The time courses of all the mentioned four measures are graphically plotted so that the dynamical interactive cardiac autonomic regulation can be investigated. Fig 1 shows the schematic diagram of the difference between the traditional method (Fig 1a) and the proposed method (Fig 1b) for HRV analysis with an example of 1-min increment. For different time increments (10 s, 30 s, 1 min, 2 min and 4 min), the same processing procedure was used for our proposed method.

## Results

### Synthetic time signal analysis

**Fig 2:**
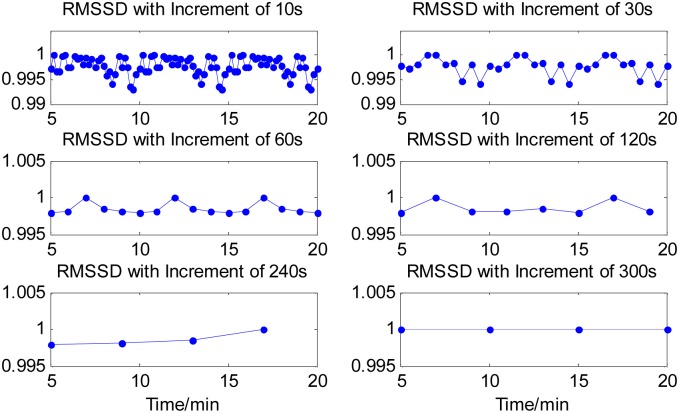
Time course of RMSSD calculated from signal segments created by sliding window with six time increments. With the time increment increasing, the time courses of the measure become smoother, resulting in losing the dynamical information. When increasing the time increment to 5min, the measure could not provide any information about the temporal dynamics in the representative synthetic signal.

**Fig 3:**
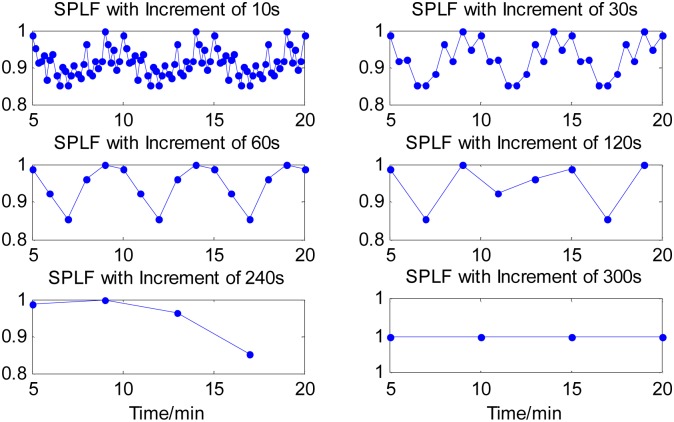
Time course of SPLF components derived from signal segments created by sliding window with six time increments. With the time increment increasing, the time courses of the measure become smoother, resulting in losing the dynamical information. When increasing the time increment to 5 min, the measure could not provide any useful information about the temporal dynamics in the representative synthetic signal.

**Fig 4:**
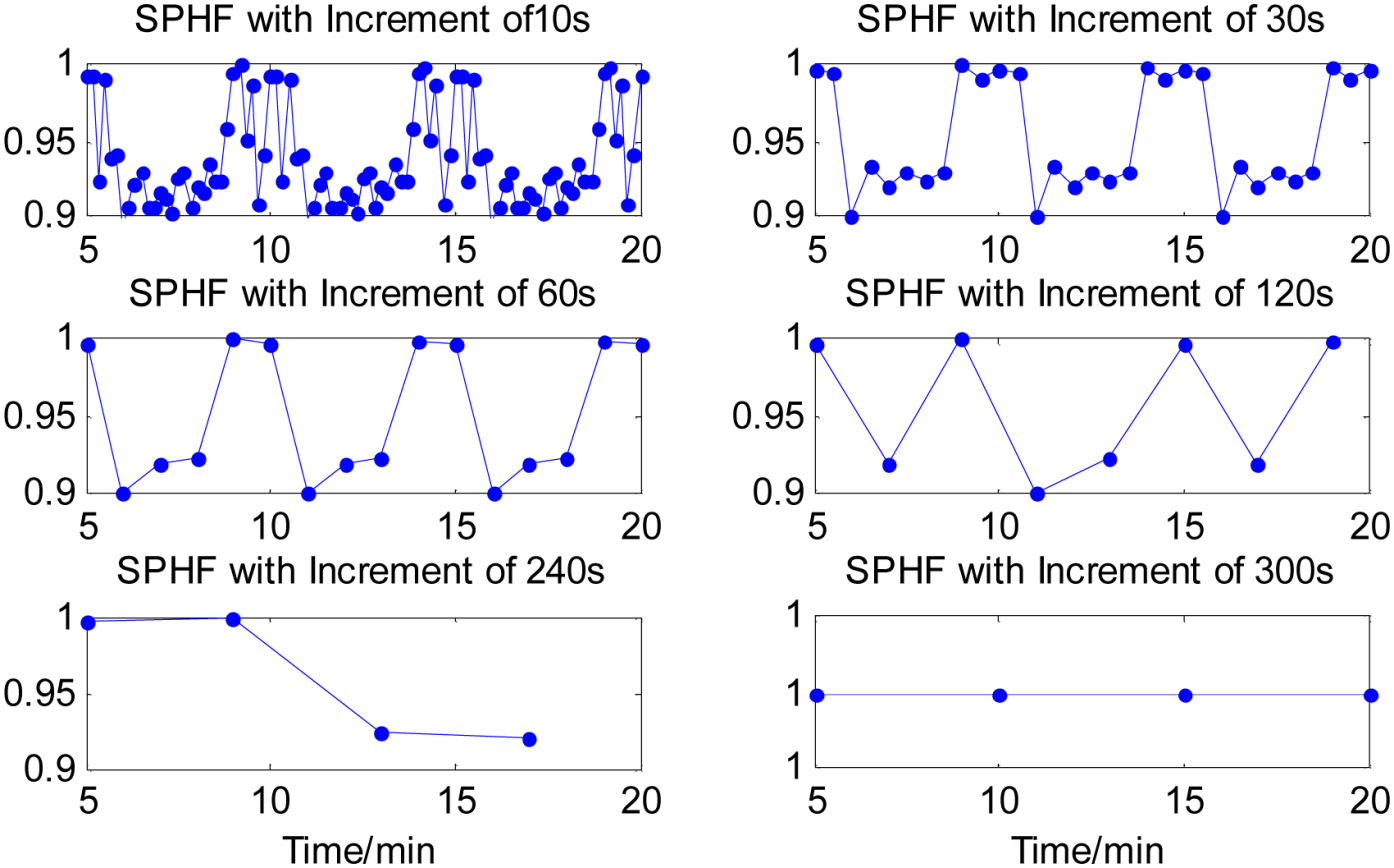
Time course of SPHF components derived from signal segments created by sliding window with six time increments. With the time increment increasing, the time courses of the measure become smoother, resulting in losing the dynamical information. When increasing the time increment to 5 min, the measure could not provide any useful information about the temporal dynamics in the representative synthetic signal.

The four time-domain and frequency-domain measurements were derived from each analysis window of the 20-min time series. The time courses of all the four measurements of the synthetic time series were computed for each of the six cases of time increments and graphically illustrated in Figs 2–4, respectively, except the measure of SDNN(t) sequence that had a constant value for the different time increments. The values of all the measures were normalized by their maxima, respectively. From Figs 2–4, it can be seen that all the three measures with a 10s time increment could provide the most detailed dynamical information on transient changes in the synthetic signal compared with other five conditions. With the time increment increasing, the time courses of the three measures become smoother, resulting in the loss of the dynamical information. When increasing the time increment to 5 min, which means there was no overlapping between two adjacent analysis windows, all the three measures could not provide any information about the temporal dynamics in the representative synthetic signal. These results suggested that using a smaller time increment could capture more dynamical information on transient changes in the synthetic signal. Note that if the time increment was too small such as 10 s, the time courses of the measures would be too indented for analyzing the dynamic changes of cardiac activities well, as shown in Figs 2–4. We knew from the Figs 2–4 that a 1-min time increment may be a good trade-off for properly capturing the dynamic information on the transient changes with a relative smooth waveform of the measures. Since the goal of this study was to evaluate the feasibility and performance of the newly proposed method in exploring temporal changes in heart rate by performing the HRV analysis in a series of consecutive short segment of ECG recordings, thus a 1-mintime increment was adopted in the rest of the present study. Obviously, for a specific application of the proposed method, if the duration of dynamic changes in cardiac activity is less than 1 minute, a smaller time increment may be needed.

### HRV analysis of Human ECG and Dog ECG

For each of the three cases of human RR-interval series, the HRV analysis was performed on a time window with a length of five minutes and increments of one and five minutes, respectively. The time courses of the four different measures were computed from each of the analysis windows and normalized with the maximum value of each measure.

**Fig 5:**
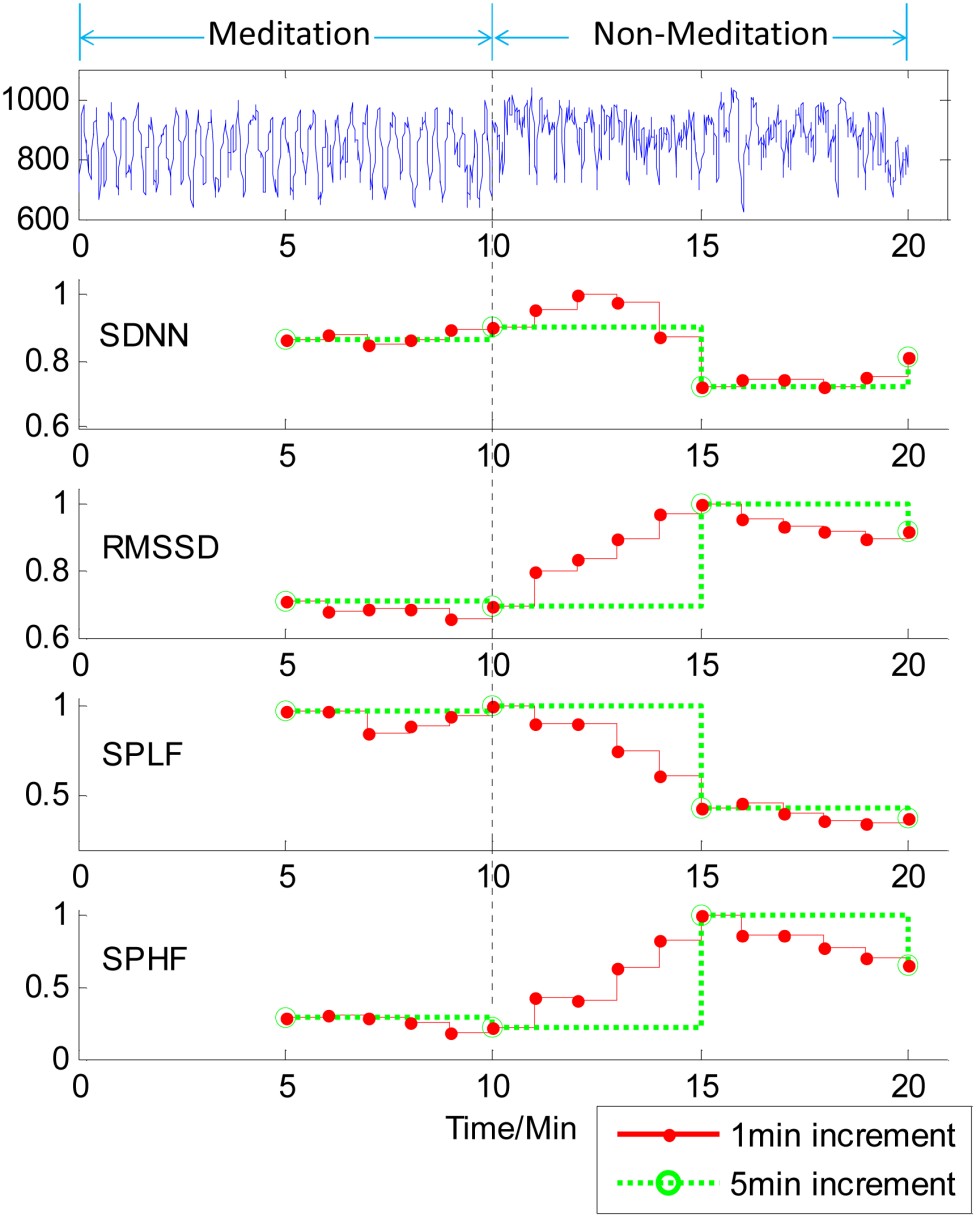
Time courses of HRV measures on human RR-interval series with1-min increment (red solid line) and 5-min increment (green dotted line). The five rows from top to bottom are the time course of RR interval series and four measures, SDNN, RMSSD, SPLF and SPHF, respectively. It can be seen that when the RR-interval data changed from meditation state to non-meditation state, all the four time courses corresponding to 1-min increment began to be obviously changed after one minute, whereas those corresponding to a 5-min increment were constant until the 5 minutes later.

For the first case of the human RR-interval series, the four time courses of HRV analysis were shown in Fig 5. It can be seen from Fig 5 that for the first 10-min RR-interval data in meditation, all the four measures were almost unchanged when using either a 1-min increment or a 5-min increment. When the process of the HRV analysis was coming into the RR-interval data in non-meditation, the four time courses corresponding to 1-min increment began to change obviously after one minute responding to the difference of meditation state, whereas those corresponding to a 5-min increment kept constant within 5 minutes. For the 1-min time increment, the four measures either decreased (SDNN and SPLF) or increased (RMSSD and SPHF) and gradually reached to new flat values with a slight oscillation. Note that all the four time courses of HRV analysis had provided similar information on delineating the dynamics of momentary HRV measurement. Thus only one measure, SPLF, was used in the HRV analysis next.

**Fig 6:**
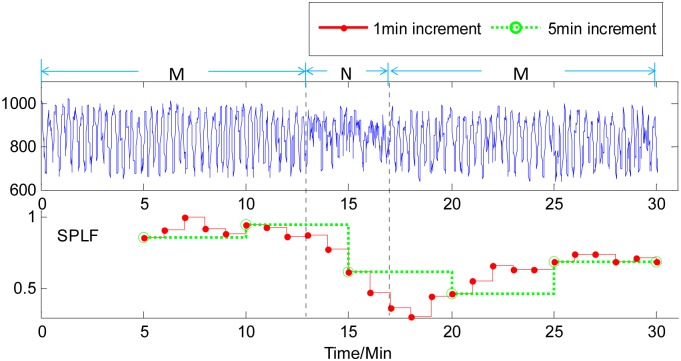
Time course of the SPLF on human RR-interval series with1-min increment (red solid line) and 5-min increment (green dotted line). M and N labelled on the signal represent Meditation and Non-meditation state, respectively. It can be seen that 1-min-increment-based HRV analysis measure gives an earlier responses to the changes of meditation states than 5-min-increment-based measure.

**Fig 7:**
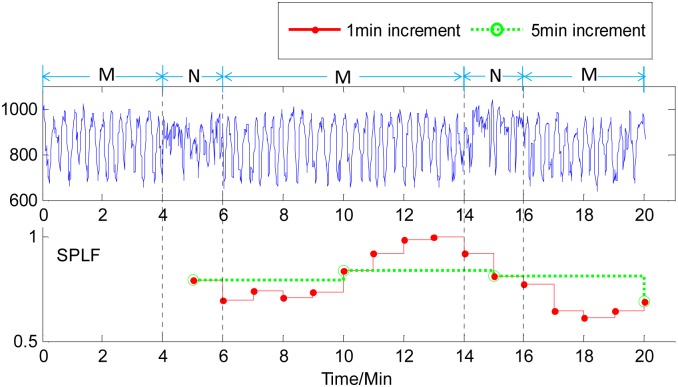
Time course of the SPLF on human RR-interval series with1-min increment (red solid line) and 5-min increment (green dotted line). M and N labelled on the signal represent Meditation and Non-meditation state, respectively. It can be seen that a 5-min-increment-based measure was hardly to capture the happenings of 2-min non-meditation state among the meditation states, 1-min-increment-based measure could clearly catch the short-time changes of meditation state.

The time courses of the measure SPLF for the HRV analysis of the second and third cases of human RR-interval series are shown in Figs 6 and 7, respectively. Similarly, for a 4-min non-meditation data in the middle of meditation data, using a 1-min increment in the HRV analysis could capture the changes of meditation states earlier than using a 5-min increment. From Fig 7, it can be observed that for the third case of human RR-interval series, the traditional HRV analysis method, i.e. using a 5-min increment herein, was hardly to capture the happenings of 2-min non-meditation state among the meditation state, while the proposed method with a 1-min increment could catch the short-time changes of meditation state clearly. In addition, it was noteworthy that the time courses of all the measures of HRV analysis with the 1-min increment could present more dynamic information of momentary heart rate variability in comparison to the 5-min increment.

**Fig 8:**
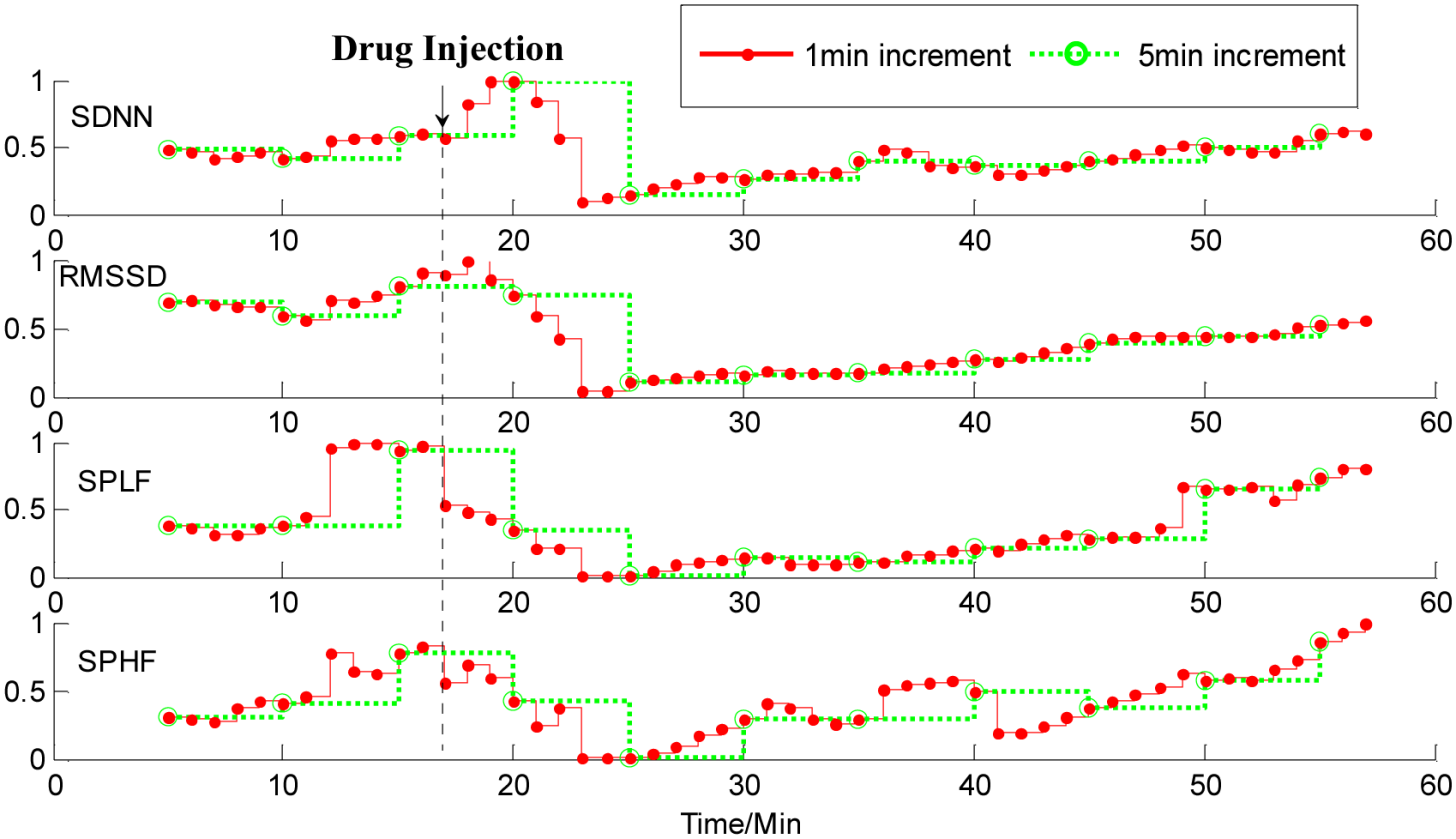
Time courses of measures of HRV analysis on ECG data from a dog with1-min increment (red solid line) and 5-min increment (green dotted line). Four rows from top to bottom correspond to the SDNN (standard deviation of the normal-to-normal intervals), RMSSD (square root of the mean squared differences of successive intervals), SPLF (power spectrum in low frequency), and SPHF (power spectrum in high frequency) components. Following the application of atropine, all the four measures of HRV analysis with 1-min increment instantly decreased and reached their minimum values within about seven minutes, and then gradually increased. With a 5-min increment, the four measures began to decrease about three minutes later after giving atropine.

Fig 8 shows the HRV analysis results on ECG data from a dog with a 1-min increment and a 5-min increment, respectively. The time of drug injection (400 mg.) is labeled by a dash vertical line on the figure. The time courses are over 60 minutes. We can see from Fig 8 that following the application of atropine, which is a blocker of parasympathetic activity, all the four measures of HRV analysis with the 1-min increment almost instantly decreased and reached their minimum values within about seven minutes, and then gradually increased. With a 5-min increment, the four measures of HRV analysis began to decrease about three minutes later after giving atropine.

## Discussion

HRV analysis has been widely accepted as the mean of determining cardiac autonomic nervous function. The HRV is measured commonly with both time-domain and frequency-domain methods [1–3, 6–12]. Conventionally, most of approaches derived from the time-domain and the frequency-domain are based on either short-term recording or long-term recording or both to implement the HRV analysis, and the Fourier transform and autoregressive modelling have been usually used for the HRV spectral analysis under the assumption of stationary system. Although these time and frequency analysis measures of HRV have provided valuable insights into the cardiac autonomic nervous function, they are limited by their inability to adequately assess transient changes in heart rate, which are associated with rapid changes in physiological status, due to all conventional measures are averaged over quite a long time. To evaluate the temporal effects of the transient change in cardiac activity on the heart rate, a method for measuring mHRV that follows these changes is required. Furthermore, the conventional frequency-domain methods of HRV analysis have fallen short in pin-pointing the non-stationary nature of HRV for robust physiological investigation.

In the present study, our goal was to perform HRV analysis based on a short-time ECG recording for continuously monitoring the temporal dynamics in heart rate. Using a smaller window for HRV analysis, the sensitivity of transient changes in detection should be higher, which has the advantage of preventing trends, transient events, biological disturbances, and so on. On the other hand, the time series for HRV analysis should be long enough to have an appropriate number of periodic events. Thus we proposed to segment the ECG recordings into a series of overlapped analysis windows and then to get the RR-interval series from each analysis window for HRV analysis. The overlapped analysis window in the proposed method had a same length of 5 minutes, which was commonly used in traditional HRV analysis, but had a time overlapped. The analysis results of the synthetic time signals suggested that a smaller time increment could capture more dynamical information on transient changes in the synthetic signal than a bigger time increment (Figs 2–4). While if the time increment was too small such as 10 s, the time courses of the three measures would be too indented. Thus in the rest of the present study, a 1-min time increment was preferred. We noted that a system of the minute-to-minute capture of HRV is offered in a commercial basis with the name of Roll Over (Cardios, São Paulo, Brazil, http://www.cardios.com.br/noticias_detalhes.asp?idNoticia=275&IdSecao=26&IdTipoNoticia=7). However, we did not get any materials and published literatures about the feasibility and performance of the proposed method in capturing the momentary changes in autonomic systems after fully searching. Thus it would be necessary to systematically investigate and report the effectiveness of the proposed HRV analysis method in delineating the dynamics of mHRV measurement before using it as a new technique.

To evaluate the performance of the proposed algorithms for measuring temporal dynamics in non-stationary RR-interval time series, human and dog ECG data were used to perform the time-domain and frequency-domain analysis. For a long-time ECG recording, the measures of HRV of each segment recording were obtained and the corresponding time courses, which reflect the trend of HRV measures over ECG recording time and likely provide the diagnostic values rather than individual data point that may be due to normal physiological fluctuations, were graphically plotted. This allows observing the dynamical evolution of heart rate. Note that it should be possible to further increase the temporal resolution by reducing the time increment. However, if the time increment is too short, the new information involved in a HRV analysis window would be less, which may lead to the indented measures of HRV. In addition, a small time increment would increase computational burdens in real-time monitoring of dynamical information of HRV. Therefore, a suitable time increment would be properly chosen according to a particular application of the proposed method.

The necessity and performance of the proposed method of short time increment have been primarily evaluated by synthetic time series. In the representative example as shown in Figs 2–4, all four measures derived from sliding windowed segments with 1-min time increment clearly demonstrate the dynamical changes in the synthetic signal, whereas all four measures from sliding windowed segments with 5-min time increment did not provide valuable information on transient changes of synthetic signal due to the averaging of these measures over 5-min signal segments that have identical patterns.

As one of possible tool of monitoring human autonomic regulation of cardiac function, the HRV dynamics has been investigated. In order to assess the performance of the proposed measures of HRV by separating data into a series of analysis windows with an increment of 1 minute, RR interval record sets acquired from healthy young adults during meditation state associated with slow breathing and non-meditation state (archived in MIT-BIT database) were used to implement the HRV analysis. The results as shown in Figs 5–7, it can be found that the proposed method delineated the dynamic process in a higher time resolution and gave a more detailed description of the dynamic changes of ANS activity compared to the traditional method. This suggested that without changing the trends of the displayed waveform, overlapped windows help us getting a detailed result by introducing of a small amount of new data each time. Animal ECG data recorded in well-control dog experiment under the application of atropine was also applied to further test the performance of the proposed method. The result showed that as the computation interval was shorter, the proposed method could catch the trends earlier than traditional method. All the results suggest that the proposed measures should be a sensitive and specific non-invasive marker for monitoring autonomic regulation of cardiac function.

## Conclusions

We have proposed a continuously monitoring system to explore temporal dynamics in heart rate by the HRV analysis using sliding window with short time increment. Using simulated non-stationary time series and real RR-interval series of human and animal ECG, we have demonstrated that the proposed measures of HRV in time-domain and time-frequency domain are able to provide more useful and detailed information in some cases when the nature of physiological modulation in heart rate changes from one short-time recording to another or heart rate has a short-time fluctuation caused by pathological intervention, as compared with the traditional measures of HRV. These pilot results obtained in the present study suggest that it would be worthy performing more investigations before the proposed method may become an important alternative tool of monitoring human autonomic regulation of cardiac function.

## Supporting Information

S1 Data The dog ECG recordings used for the HRV analysis. (TXT)
